# Isolated Central Hypothyroidism in an Adolescent With Narcolepsy

**DOI:** 10.7759/cureus.8496

**Published:** 2020-06-07

**Authors:** Sana Alhajri, Haesoon Lee, Abdul Hasan Siddiqui, Sheila Perez-Colon

**Affiliations:** 1 Pediatrics, State University of New York Downstate Medical Center, Brooklyn, USA; 2 Pediatric Pulmonology, State University of New York Downstate Medical Center, Brooklyn, USA; 3 Pulmonary and Critical Care Medicine, University of Illinois at Urbana-Champaign, Champaign, USA; 4 Pediatric Endocrinology, State University of New York Downstate Medical Center, Brooklyn, USA

**Keywords:** central hypothyroidism, narcolepsy, somnolence, obesity, excessive daytime sleepiness

## Abstract

Isolated central hypothyroidism (ICH) and narcolepsy are conditions rarely seen in the pediatric population which are usually characterized by delayed diagnosis and treatment due to their variable presentation and subclinical onset. We describe an unusual case of an adolescent male diagnosed with narcolepsy and central hypothyroidism. A 15-year-old obese boy presented with the complaint of excessive daytime sleepiness, fatigue, and snoring. Obstructive sleep apnea (OSA) was initially suspected as the underlying cause, but the sleep study was negative for OSA. However, the multiple sleep latency test was consistent with narcolepsy without cataplexy. He was then started on modafinil, but his symptoms persisted. Thyroid function tests were performed that were consistent with ICH. Thyroid replacement therapy was initiated with subsequent improvement in symptoms. A theoretical association exists between narcolepsy and ICH due to the involvement of the hypothalamus and pituitary gland. Nevertheless, clinical association, as seen in our case, is rare. Central hypothyroidism is a known etiology leading to fatigue and sleepiness. Narcolepsy without cataplexy can have overlapping symptoms with hypothyroidism, as seen in our patient. The presence of narcolepsy should prompt screening for hypothyroidism in appropriate clinical settings.

## Introduction

Although recent studies have addressed the association of narcolepsy with other endocrine abnormalities, isolated central hypothyroidism (ICH) is rarely described in the pediatric population with narcolepsy. ICH is usually characterized by delayed diagnosis and treatment due to its variable presentation and subclinical onset [1]. It is often missed in the newborn period due to thyroid-stimulating hormone (TSH)-based screening [2]. Nevertheless, undiagnosed central hypothyroidism can be detrimental to cognition and metabolism. We present a case of ICH in a patient with narcolepsy with no significant improvement of symptoms on modafinil.

## Case presentation

A 15-year-old obese boy was evaluated for excessive daytime sleepiness and snoring. He denied any cataplexy attacks or hypnopompic or hypnagogic hallucinations. His medical history was negative, except for obesity (body mass index (BMI): 42 kilograms/meter^2^), and he was not on any medication. He denied any family history of similar presentations or sleep disorders. He was seen at the Pediatric Pulmonology Clinic and was scheduled for an in-lab sleep study. The study showed an overall apnea-hypopnea index (AHI) of 2.4 (< 5 is normal), ruling out obstructive sleep apnea (OSA) and central sleep apnea. Due to his persistent excessive daytime sleepiness, a multiple sleep latency test (MSLT) was scheduled. The MSLT revealed pathological daytime sleepiness with a sleep latency < 8 mins on more than two occasions, along with four sleep-onset rapid eye movement (REM) periods. The findings of the MSLT were suggestive of a diagnosis of narcolepsy. 

The patient was then started on modafinil for the treatment of narcolepsy. Despite the pharmacologic therapy for narcolepsy, his symptoms persisted. For his obesity and persistent daytime sleepiness, thyroid function tests (TFTs) were performed which revealed a TSH level of 0.4 uIU/mL (range: 0.35 - 4.7 uIU/mL) (inappropriately normal) and a free T4 of 0.59 ng/dL (low) (range: 0.7 - 1.8 ng/dL) with negative thyroglobulin antibodies and anti-peroxidase antibodies (Table 1). Repeated TFTs confirmed central hypothyroidism. Magnetic resonance imaging (MRI) of the brain revealed no significant abnormality related to the brain or the pituitary gland. The remainder of the pituitary hormones were normal, except for a baseline cortisol level of 4.3 μg/dL (normal range: 4 - 22 μg/dL). A low-dose adrenocorticotropic hormone (ACTH) stimulation test (1 mcg IV) was done with cortisol levels checked at 30 and 60-minute intervals; the results were 18.2 μg/dL and 10.2 μg/dL, respectively. A high-dose ACTH stimulation test (250 mcg IV) was also done with cortisol levels checked at 30 and 60-minute intervals; the results were 20.4 μg/dL and 24.2 μg/dL, respectively. Based on the symptoms and TFT results, isolated central hypothyroidism was diagnosed. The patient was subsequently started on thyroid replacement therapy while continuing with the modafinil therapy. On follow-up visits, there was an improvement in weight and sleep symptoms.

**Table 1:** Laboratory Studies * Six months after treatment with levothyroxine, 75 mcg PO once daily FT4: free thyroxine; TG:  thyroglobulin; TG Ab: thyroglobulin antibody; TPO Ab: thyroid peroxidase antibody; TSH: thyroid-stimulating hormone; TSI: thyroid-stimulating immunoglobulin; TT3: total triiodothyronine

	FT4	TSH	TT3	TG	TPO Ab	TG Ab	TSI
Reference Values	0.7 - 1.8 ng/dL	0.35 - 4.7 uIU/mL	59 - 174 ng/dL	2 - 35 ng/mL	0 - 26 IU/ml	0.0 - 0.9 IU/ml	0 - 139%
Baseline	0.59	0.4	60	16.2	< 26	< 1	
*Post-treatment	0.81	< 0.05	75				24

## Discussion

Our case highlights the similar aspects of two different conditions, narcolepsy without cataplexy and isolated central hypothyroidism, which are not only rare but also known to have overlapping clinical features. Both of these conditions are challenging to diagnose and treat in the pediatric population. Moreover, both conditions are often associated with a delayed diagnosis and, at times, misdiagnosis [1-2]. Bartels et al. described that 14% of patients diagnosed and treated for hypothyroidism were found to have narcolepsy [3]. In an adult study, Kok et al. demonstrated in their study of seven patients and seven controls that patients with narcolepsy can have concomitant thyrotropin deficiency [4]. The study highlighted that excessive sleepiness associated with narcolepsy might have an inhibitory effect on the release of TSH which they have demonstrated by showing low TSH levels in subjects who slept longer than controls. However, they failed to explain the underlying mechanisms. Deficiency of leptin hormones, i.e., hyperleptinemia, seen in narcoleptic patients has been described as a possible cause of thyrotropin abnormalities and resultant hypothyroidism as was described in a few animal studies [5]. Other studies have also shown the association of these two conditions with dopaminergic and noradrenergic pathways that play a crucial role in thyroid regulation [6].

In the late 1990s, hypocretin orexin (a hypothalamic neurotransmitter) deficiency was found to play a central role in the pathogenesis of narcolepsy. Since then, multiple studies have demonstrated a pathogenic link between narcolepsy and various endocrine disorders, such as thyroid, adrenal, insulin, and growth hormone-related abnormalities [7].

Narcolepsy is a neurologic disorder characterized by excessive daytime sleepiness (EDS), hypnogogic hallucinations, with or without cataplexy, and sleep paralysis [4]. Gelineau first described the term narcolepsy in 1880 [8]. The prevalence of narcolepsy is estimated to be in the range of 20 - 60 of every 100,000 subjects in the United States (US) and Europe [5]. Depending on the presence of hypocretin deficiency and cataplexic attacks, narcolepsy is broadly divided into two categories. Type 1 is characterized by EDS, hypnogogic hallucinations, sleep paralysis, and cataplexy attacks with hypocretin deficiency, whereas Type 2 is characterized by EDS, hypnogogic hallucinations, sleep paralysis without cataplexic attacks, and normal hypocretin [9]. The disease usually begins between the ages of 10 and 20 years with the sudden onset of persistent daytime sleepiness, although it can also develop gradually [8]. Genetic research has led to the identification of specific human lymphocyte antigen (HLA) alleles, DQB1∗0602, and DRBI∗1501, which predisposes for the disorder. Based on its association to HLA, it is often presumed to have autoimmune pathophysiology that has not yet been proven because of the lack of demonstration of an autoantibody specific to the disease and the presence of typical inflammatory markers, such as erythrocyte sedimentation rate (ESR), C-reactive protein (CRP), immunoglobulin levels, and complement factors [5].

Establishing a diagnosis of narcolepsy can be challenging in pediatric patients, given the variability in clinical presentation, the limited descriptive ability of the child, and the reliability of the symptoms described by the parents. Quite often, the diagnosis is made only after serious problems have arisen, such as declining grades at school and poor performance at work, among others. The current diagnosis is based primarily on the clinical picture with the assistance of sleep recordings and HLA typing [9]. Cerebrospinal fluid (CSF) studies demonstrate low to absent hypocretin levels are sensitive for Type 1 narcolepsy, but this test is not readily available and is not always needed to start the patient on therapy for narcolepsy.

In general, patients with narcolepsy are considered to be at risk for obesity and diabetes as a deficiency of hypocretin leads to the dysregulation of energy homeostasis, endocrine systems, and sleep (4). This condition is usually treated with behavioral and pharmacological therapy. Inadequate sleep, untreated sleep apnea, periodic limb movement during sleep, and sedating medications should be excluded when narcolepsy is considered. Neurostimulants, such as modafinil, amphetamines, and dextroamphetamine, are used for the EDS, and selective serotonin reuptake inhibitors, such as fluoxetine, citalopram, and sertraline, are used for the treatment of cataplexy.

Central hypothyroidism (CH), on the other hand, is a rare cause of hypothyroidism, generally due to either pituitary or hypothalamic defects. The classic biochemical abnormality seen in these patients includes a low serum level of circulating thyroxine (T4) concomitant with an inappropriately normal or low level of thyrotropin (TSH). By its etiology, it is possible to distinguish acquired and hereditary forms. Hereditary CH can be isolated or associated with combined pituitary hormone deficiency (CPHD). In the former case, alterations of only two genes, TSH beta and the thyrotropin-releasing hormone (TRH) receptor, have been described as responsible for the disorder [10]. The clinical consequences of CH in life vary greatly depending on the etiology, the severity of the thyroid impairment, the extent of the associated hormone deficiencies, and the age of the patient at the time of the onset of the disease. In general, acquired CH is less severe than the congenital form because of the constitutive activity of the wild-type TSH-receptor. Symptoms and signs of thyroid insufficiency are usually milder than those of primary hypothyroidism, and the goiter is always absent. In CPHD, most patients have other endocrine manifestations of the disease (growth failure, delayed puberty, adrenal insufficiency, or diabetes insipidus) that lead them to seek medical attention before hypothyroidism becomes apparent. Early diagnosis of the congenital form by neonatal screening for hypothyroidism is strongly recommended to avoid cretinism. Replacement therapy with L-thyroxine has to be established as soon as possible. A TRH test is not required to make a diagnosis of central hypothyroidism in whom other biochemical or neuroradiological hypothalamic-pituitary (H-P) abnormalities have been demonstrated, particularly in light of the occasional discomfort to the patients resulting from the administration of TRH (nausea and flushing) [11].

Though anecdotally and in pilot studies, a link between narcolepsy and TRH abnormality has been demonstrated in otherwise clinically euthyroid patients; isolated CH in a patient with Type 2 narcolepsy is rarely seen. Our case highlights the need for clinicians to be aware of the various presentation and abnormalities associated with narcolepsy in pediatric patients. We recommend that patients diagnosed with narcolepsy should have TFT done routinely. Moreover, when symptoms don’t improve as expected on narcolepsy medications, TFT is warranted.

The abstract of this case report was accepted for poster presentation at the American Thyroid Association (ATA) Annual Meeting in September 2016 in Denver, Colorado, and the abstract was published in the ATA journal, Thyroid, annual meeting supplement [12]. 

## Conclusions

Isolated central hypothyroidism and narcolepsy in the pediatric age group are often found to be subclinical and are usually associated with delayed diagnosis and misdiagnosis. When a patient with EDS and narcolepsy fails to respond adequately to a neurotransmitter, such as modafinil, physicians should look for other causes of EDS. Hence, a thorough knowledge of various presentations and associations is crucial to prevent adverse outcomes and sequelae later in life. Educational interventions should be made among healthcare workers and the general population about these two conditions for early detection and treatment. In addition, the presence of narcolepsy should prompt screening for central hypothyroidism in the appropriate clinical settings.
